# Visual read of [F‐18]florquinitau PET that includes and extends beyond the mesial temporal lobe is associated with increased plasma pTau217 and cognitive decline in a cohort that is enriched with risk for Alzheimer's disease

**DOI:** 10.1002/alz.14406

**Published:** 2024-11-19

**Authors:** Ramiro Eduardo Rea Reyes, Karly A. Cody, Rachael E. Wilson, Henrik Zetterberg, Nathaniel A. Chin, Erin M. Jonaitis, Melissa Bahr, Olivia Mandel, Madilynn Wintlend, Barbara B. Bendlin, Ozioma C. Okonkwo, Lindsay R. Clark, Matt Zammit, Sanjay Asthana, Bradley T. Christian, Tobey J. Betthauser, Laura Eisenmenger, Rebecca E. Langhough, Sterling C. Johnson

**Affiliations:** ^1^ Wisconsin Alzheimer's Disease Research Center University of Wisconsin School of Medicine and Public Health Madison Wisconsin USA; ^2^ Department of Neurology and Neurological Sciences Stanford University Palo Alto California USA; ^3^ Department of Psychiatry and Neurochemistry Institute of Neuroscience and Physiology the Sahlgrenska Academy at the University of Gothenburg Mölndal Sweden; ^4^ Clinical Neurochemistry Laboratory Sahlgrenska University Hospital Gothenburg Sweden; ^5^ Department of Neurodegenerative Disease UCL Institute of Neurology, Queen Square London UK; ^6^ UK Dementia Research Institute at UCL London UK; ^7^ Hong Kong Center for Neurodegenerative Diseases Clear Water Bay, Science Park Hong Kong China; ^8^ Wisconsin Alzheimer's Institute University of Wisconsin School of Medicine and Public Health Madison Wisconsin USA; ^9^ Geriatric Research Education and Clinical Center William S. Middleton Memorial Veterans Hospital Madison Wisconsin USA; ^10^ Department of Medical Physics University of Wisconsin School of Medicine and Public Health Madison Wisconsin USA; ^11^ Waisman Center University of Wisconsin‐Madison Madison Wisconsin USA; ^12^ Department of Radiology University of Wisconsin School of Medicine and Public Health Madison Wisconsin USA

**Keywords:** cognitive composite, cognitive trajectories, PET qualitative assessment, PET visual read, plasma biomarkers, pTau217

## Abstract

**INTRODUCTION:**

Patterns of signal from tau positron emission tomography (tau‐PET) confined to the medial temporal lobe (MTL) or extended into the neocortex may be relevant for Alzheimer's disease (AD) research if they are linked to differential biomarker levels and cognitive decline.

**METHODS:**

Visual assessment of Tau‐PET [F‐18]florquinitau (FQT) exams from 728 initially non‐demented older adults yielded four uptake groups: tau‐negative (T−), MTL‐only (T+_MTL_), neocortex‐only (T+_Neo_), or both (T+_MTL&Neo_). Mixed effects models assessed group differences in retrospective cognitive and plasma pTau217 trajectories.

**RESULTS:**

T+_MTL&Neo_ was the most common T+ group (*n* = 97; 93% A+) and exhibited the sharpest worsening in cognitive and pTau217 trajectories before tau PET.

**DISCUSSION:**

The T+_MTL&Neo_ category represents an intermediate to advanced stage of AD preceded by rising ptau217 and progressive cognitive decline. The pTau217 finding suggests that A+, T+ in MTL or neocortex could represent early AD stages, with a higher likelihood of progressing to more advanced stages.

**Highlights:**

- Visual assessments of Tau‐PET FQT revealed four distinct uptake groups: tau‐negative (T−), MTL‐only (T+_MTL_), neocortex‐only (T+_Neo_), or both (T+_MTL&Neo_).
- Amyloid positive participants in the T+_MTL_ and T+_MTL&Neo_ categories showed a retrospectively faster decline in their cognitive trajectories, and a sharper increase in pTau217 levels in plasma, compared to T−.
- The T+_MTL&Neo_ group displayed sharper trajectories compared with the other Tau positive groups in both their cognitive scores and pTau217 plasma levels.
- Our results suggest that participants with Tau present in both MTL and neocortex represent an intermediate to advanced stage of AD, whereas participants with signals confined to either MTL or neocortex could represent earlier AD stages.

## 1 BACKGROUND

The tau PET imaging radiotracer [F‐18]florquinitau (FQT; also known as [F‐18]MK‐6240)^1^, ^2^, ^3^ has a high affinity for fibrillar tau tangles^2^, ^3^ and the spatial progression of its brain retention follows a characteristic pattern resembling Braak stages.^4^, ^5^, ^6^ We and others have found strong relationships between quantitative FQT signal and cognitive stage as well as cognitive change among initially non‐symptomatic^6^, ^7^ and symptomatic individuals.^7^, ^8^, ^9^ While these quantitative measures have led to major research insights, their generalizability to less quantitative clinical settings remains unknown.

Recently a framework for visually rating FQT images was reported,^10^ which involves specifying when the signal on the exam is confined to the medial temporal lobe (MTL), the most common originating region for tau proteinopathy in AD, and when the signal also involves the neocortex. Such a framework may be advantageous in certain contexts for qualitatively describing the extent of tau disease burden. In the clinical setting, FQT visual reads could have high utility if they are shown to be associated with clinically relevant disease indicators of amyloid pathology, plasma phosphorylated tau (pTau) concentration, and cognitive decline, as was previously shown for [F‐18]flortaucipir.^11^ A visual read may also add value in cases where standard reporter regions of interest (ROIs) do not spatially coincide with the preponderance of the signal coming from an individual person's exam since tau spatial patterns may vary^12^, ^13^ within and between regions from person to person and, thus, may not always conform to predesignated ROIs.

This study addresses a set of knowledge gaps that may be of broad interest pertaining to whether patterns of tau signal confined to or extending beyond the MTL into the neocortex are differentially associated with amyloid PET positivity, retrospective plasma pTau217 trajectories, and most importantly with preclinical cognitive decline trajectories before tau scan. To address these points, we adapted the four‐category FQT visual rating system^10^ and applied it to a large single‐site two‐cohort series.

## 2 METHODS

### 2.1 Participants

The sample for the analysis was selected from an initial pool of *n* = 770 possible participants (535 from the Wisconsin Registry for Alzheimer's Prevention [WRAP] and 235 from the Wisconsin Alzheimer's Disease Research Center [WADRC]) as shown in the consort diagram in the Figure S1. We first limited the inclusion based on: (1) a non‐dementia cognitive status at their first applicable prior cognitive evaluation of either mild cognitive impairment (MCI; 37(4.90%)) or cognitively unimpaired (CU; 714 (95.10%)); (2) available qualitative visual reads for amyloid and tau status from PET exams that were not more than 3 years apart from each other. For the analysis of cognitive trajectories, we only included participants with available scores from a modified three‐test Preclinical Alzheimer's Cognitive Composite (PACC3), and we limited the observations included in the analysis to those that occurred at most 3 years after the most recent FQT PET (*n* = 728, see Table 1). Similarly, for the analysis examining pTau217 trajectories by tau group, we limited the sample to participants who had available plasma pTau217 measurements up to 3 years after the most recent FQT PET (*n* = 693). The rationale for the 3‐year interval was to maximize inclusion of data from participants who volunteered for multiple procedures while also allowing latitude due to uncontrollable factors, chiefly the coronavirus disease 2019 (COVID‐19) pandemic stoppage that affected cognitive and blood collection concurrent to PET.

**TABLE 1 alz14406-tbl-0001:** Sample characteristics of those included in the cognitive analysis, overall and by T group.

Variable	Overall	T−	T+_Neo_	T+_MTL_	T+_MTL&Neo_	*p‐*value
Cognitive analysis, *n* (%)	728	573 (78.7%)	25 (3.4%)	33 (4.5%)	97 (13.3%)	
Race, *n* (%)						
American Indian or Alaskan native	21 (2.9%)					
Asian	1 (0.1%)					
Black or African American	52 (7.1%)					
Other	3 (0.4%)					
White, Hispanic	7 (1.0%)					
White, non‐Hispanic	644 (88.5%)					
Age at FQT scan	67.9 (7.4)	66.8 (7.4)	71.1 (5.6)^b^	73.1 (7.3)^b^	71.6 (6.1)^b^	**< 0.001**
Age at Aβ scan	66.1 (7.9)	65.0 (7.8)	69.6 (7.1)^b^	72.0 (8.0)^b^	69.7 (6.7)^b^	**< 0.001**
A+, *n* (%)	221 (30.4%)	95 (16.6%)	14 (56.0%)^b^	22 (66.7%)^b^	90 (92.8%)^b^	**< 0.001**
Baseline amyloid centiloids, median [Q1, Q3]	5.94 [0.19, 17.88]	3.38 [−0.66, 9.80]	10.16 [0.80, 35.08]	26.05 [9.97, 78.26]	75.90 [37.68, 105.02]^c^	**< 0.001**
Female, *n* (%)	504 (69.2%)	398 (69.5%)	14 (56.0%)	23 (69.7%)	69 (71.1%)	= 0.525
BA or more education, *n* (%)	502 (69.0%)	397 (69.2%)	16 (64.00%)	22 (66.7%)	67 (69.1%)	= 0.941
Age at baseline	59.6 (7.5)	58.6 (7.1)	61.6 (7.7)	64.1 (7.8)^b^	63.7 (7.7)^b^	**< 0.001**
Years of PACC follow‐up	8.7 (4.2)	8.7 (4.2)	9.9 (3.8)	8.9 (4.3)	8.4 (4.7)	= 0.442
Practice, median [Q1, Q3]	5.0 [4.0, 6.0]	5.0 [4.0, 6.0]	5.0 [4.0, 6.0]	5.0 [4.0, 6.0]	5.0 [4.0, 6.0]	= 0.666
Years between the last PACC and FQT scan	0.8 (2.0)	0.8 (2.0)	0.9 (2.1)	1.2 (2.9)	0.8 (2.0)	= 0.695
Baseline PACC	−0.01 (1.10)	0.10 (0.96)	−0.22 (1.10)	−0.13 (1.29)	−0.61 (1.55)^b^	**< 0.001**
Adjusted baseline PACC (SEM)^a^		−0.15 (0.05)	−0.21 (0.19)	−0.06 (0.17)	−0.57 (0.10)^b^	**< 0.001**
CU at baseline PACC, *n* (%)	694 (95.3%)	562 (98.1%)	24 (96.0%)	30 (90.9%)	78 (80.4%)^b^	**< 0.001**
CU at latest PACC, *n* (%)	640 (87.9%)	546 (95.3%)	20 (80.0%)	28 (84.8%)^b^	46 (47.4%)^c^	**< 0.001**

Abbreviations: BA, bachelor's degree; CU, cognitively unimpaired; FQT, [F‐18]florquinitau; PACC, Preclinical Alzheimer's Cognitive Composite.

^a^ We show the marginal effects with covariates set as follows: age = 65, education = less than BA, sex = female.

^b^ Different to T−;.

^c^ Different to all groups.

### 2.2 Imaging methods

Participants underwent [F‐18]FQT and [C‐11]Pittsburgh compound B (PiB) PET imaging and, in most cases, also underwent T1‐weighted magnetic resonance imaging (MRI) at the University of Wisconsin‐Madison medical campus. Detailed methods for radioligand synthesis and PET and MRI acquisition, processing, and quantification, and analysis were implemented as reported previously.^1^, ^14^, ^15^ Briefly, MK‐6240 involved a 20‐min imaging window in four 5‐min frames starting at 70 min post‐injection of 185–370 MBq. The start of the FQT acquisition at 70 min was chosen to minimize the relative portion of PET signal resulting from off‐target binding in the skull or meninges.^1^ The images frames were later realigned to overcome bulk motion, co‐registered to a T1‐weighted three‐dimensional MRI, and then summed and scaled to the inferior cerebellum gray as described previously. PiB imaging was acquired dynamically 0–70 min post‐injection of 555 MBq target dose from which distribution volume ratio (DVR) images were created using the cerebellum gray matter as the reference region. When a full dynamic series was not possible (not common), the 50–70 min post‐injection time window was acquired in 5‐min frames, and standardized uptake value ratio (SUVR) was created from the frame‐realigned summed image using the cerebellum gray matter as the reference region.

The Shuping et al. method^10^ was adapted as follows: FQT (SUVR; 70–90 min) images were derived and overlaid on coregistered T1‐weighted MRI, and scaled from 0 to 2.5 to assess tau burden by expert readers (S.C.J. and L.E.). Specifically, tau burden was rated visually for presence or absence in each of the two search areas (the MTL and neocortex; details in next paragraph). Both expert raters independently rated a subset of 30 cases to assess the reliability of visual ratings. The inter‐rater reliability Kappa statistics for the categories of T+_MTL_ and T+_Neo_ were *K* = 0.87 (28/30 agreement) and 1.0 (30/30 agreement), respectively. Subsequent scans were reviewed by one of the two expert readers (S.C.J.). Visual ratings on these categories were then combined to create four overall FQT tau status categories for each scan: T− (negative in both MTL and neocortex); T+_MTL_ (positive in the MTL and negative in the neocortex); T+_Neo_ (positive in the neocortex and negative in the MTL); and T+_MTL&Neo_ (positive in both regions). We assigned the participant's tau status based on the most recent visual read available from them.

**RESEARCH IN CONTEXT**

1. **Systematic review**: We conducted a thorough search of the scientific literature to explore the current state of the relationship between positron emission tomography (PET) image assessments in Alzheimer's disease (AD), blood‐based biomarkers, and cognition. Our review identified several studies demonstrating correlations between quantitative PET signals and cognitive changes. We also noted recent advances in the qualitative assessment of PET images, based on expert visual readings. Integrating these visual assessments with blood biomarker levels and cognitive scores, similar to the approach used for quantitative methods, could be particularly beneficial when the brain regions assessed quantitatively do not align with the areas generating the PET signal.
2. **Interpretation**: Our study identified distinct categories based on visual assessments of PET images using the tau radiotracer FQT. Participants presenting tau signal large enough to deem a positive rating in the MTL or in both MTL and neocortex showed a sharper declining in their retrospective cognitive scores and a more pronounced increase in plasma pTau217 levels compared to those without tau signal in these regions. Moreover, participants with tau signals in both the MTL and neocortex displayed steeper trajectories in cognitive decline and biomarker increase compared to those with signals in either the MTL or neocortex alone. Finally, we showed how these trajectories are present only in participants who also have a positive visual read for amyloid in their brains.
3. **Future directions**: Future research should aim to refine the visual read framework to distinguish signals in different neocortical areas and establish stronger links to other biomarkers for non‐AD dementias. Additionally, future studies should investigate the generalizability of these patterns across broader demographic groups.

**FIGURE 1:**
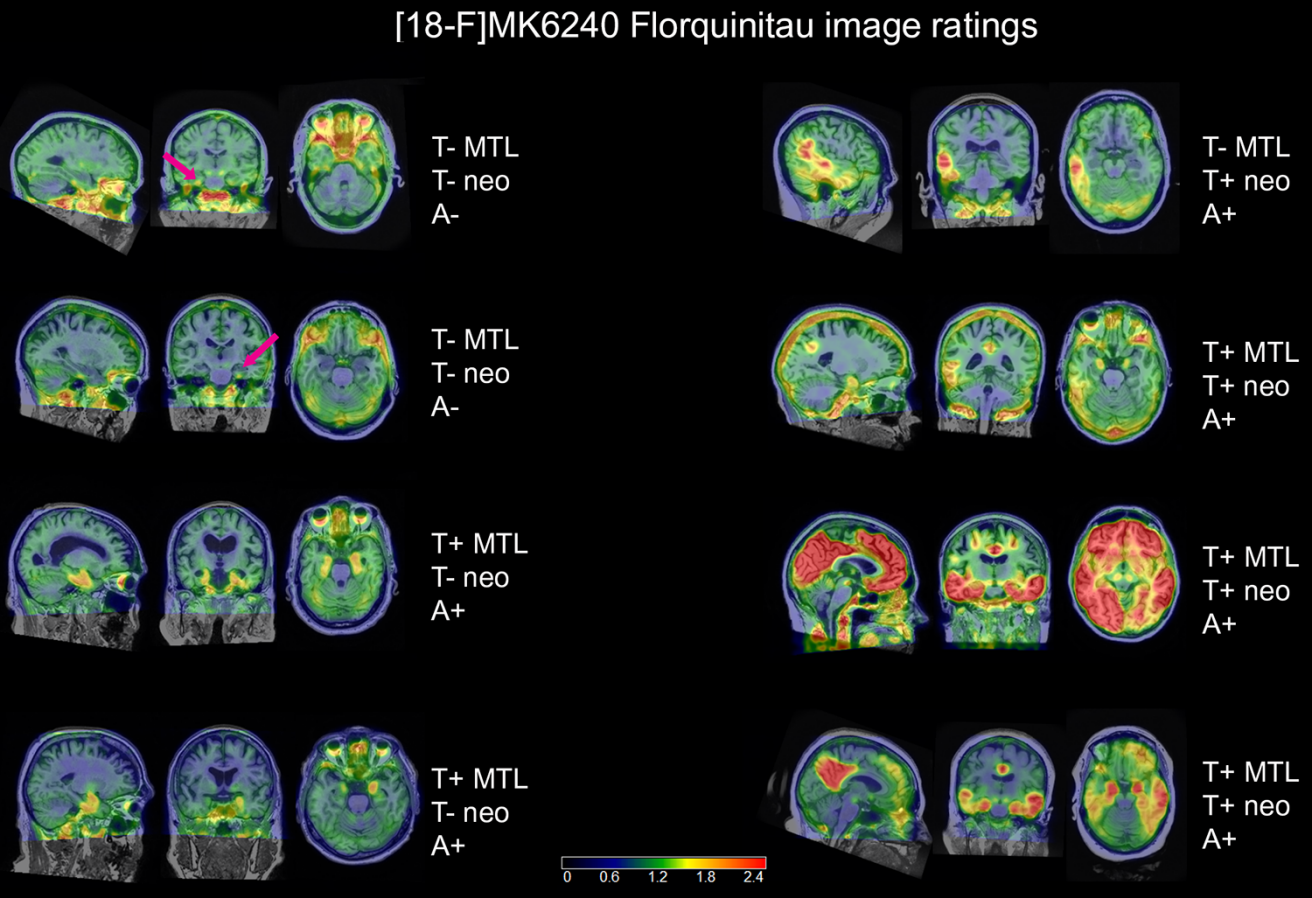
Examples of FQT PET exams in each of the tau rating categories. In each case, the standardized uptake value ratio is overlaid on a co‐registered T1‐weighted MRI and is shown in sagittal, coronal, and axial views at informative slice locations. The arrow in the first two cases points to an area of subtle punctate MTL signal that was considered tau negative. The ratings for tau PET in the MTL and neo and for amyloid PET are shown to the right of each case. FQT, [F‐18]florquinitau; MRI, magnetic resonance imaging; MTL, medial temporal lobe; neo, neocortex; PET, positron emission tomography.

**FIGURE 2:**
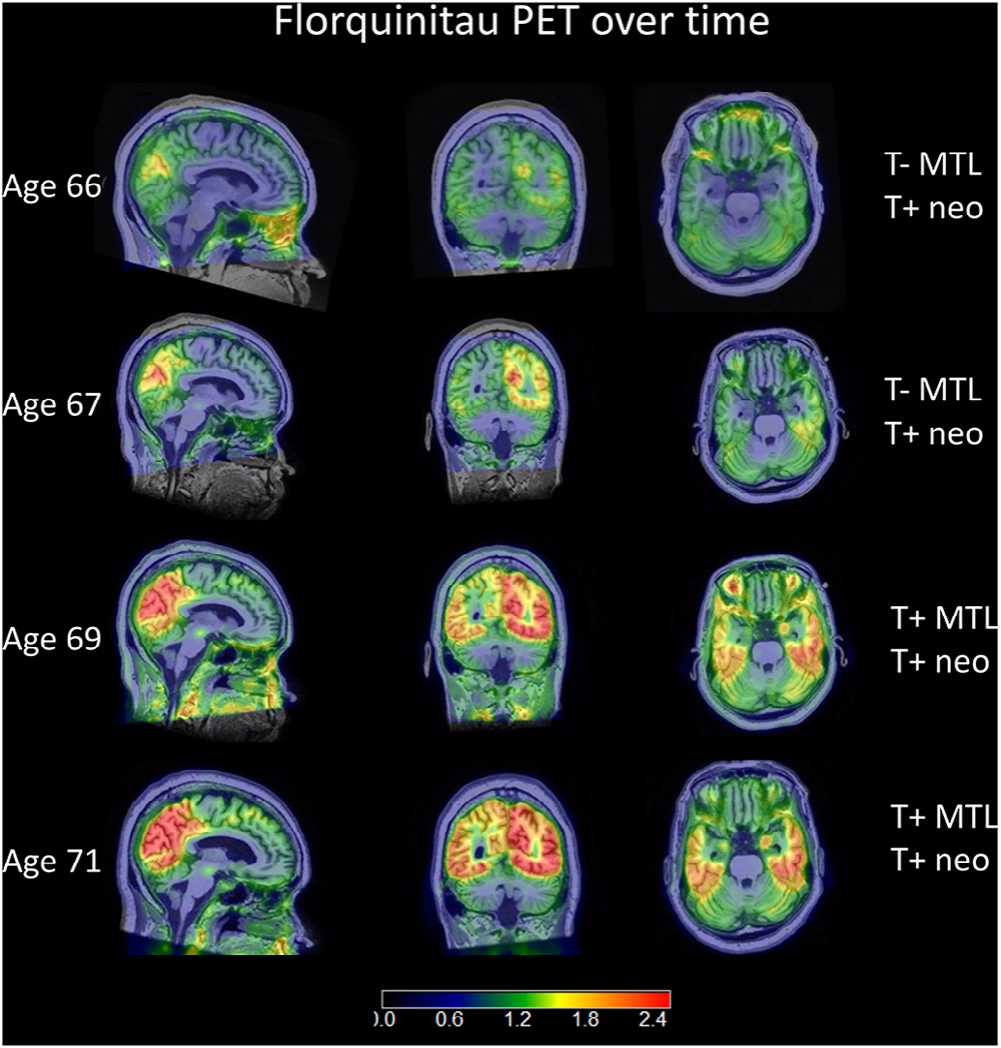
Case example of neocortical tau progression. This individual's APOE result was e3/3 and they were already amyloid positive at age 61 as observed by a PiB scan. The first FQT scan at age 66 demonstrated tau signal limited to the neocortical area of the precuneus unilaterally. Subsequently, patterns of predominant parieto‐occipital tau burden suggestive of the posterior cortical atrophy subtype of Alzheimer's disease revealed itself, though, by the third FQT exam, the MTL was also positive. APOE, apolipoprotein E; FQT, [F‐18]florquinitau; MTL, medial temporal lobe; PiB, Pittsburgh compound B.

Further details on the tau ratings: If present, spill‐in from extra‐axial tissue was not rated positive. To investigate the source of signal in such cases, the rater had the flexibility of increasing the scale range. For all exams, images were classified as tau negative or tau positive for the MTL (T+_MTL_) where the search region included the entorhinal cortex and surrounding anterior parahippocampal gyrus, amygdala, or hippocampus (which was rare). To be considered T+_MTL_, the signal needed to have a quality[Table alz14406-tbl-0001] of expansion medially or antero‐posteriorly beyond a small confined focal locus. The reason is that very small loci may be of uncertain origin and of uncertain interpretation. The neocortex tau rating: Images were classified as tau negative or positive for the neocortex (T+_Neo_) in a broad search region that included any cortical areas beyond the MTL. A categorical rating of T+_MTL&Neo_ was derived for cases in which both the MTL and the neocortex were rated T+. Example scans with these ratings are shown in Figure 1. The case in Figure 2 started as a single subtle posterior neocortical area and progressed over four exams at approximate 2‐year intervals to include the MTL and most of the posterior neocortex.

Amyloid positivity status was assessed qualitatively with visual read as positive or negative, with a positive rating requiring substantial cortical gray matter signal in one or more of medial, lateral, superior, or ventral lobe segments from any of the parietal, temporal, frontal, or occipital lobes, whether unilaterally or bilaterally observed. Within the negative category, a label of indeterminant was occasionally assigned when the signal was not of sufficient intensity or extent to be convincingly positive; these were grouped with amyloid negative in our analyses. As with the tau rating, the scaled DVR image (or SUVR when full dynamic data were not available) was overlaid on the coregistered T1‐weighted volume when available and the scale range set from 0 to 2.5 and visualized with the actc colormap in mricron. In addition to the visual rating, the amyloid burden was further quantified for descriptive purposes with a DVR cortical composite metric and centiloid values as previously described.^14^ The relationship of a positive visual read outcome to quantitative DVR and centiloid corresponded to a DVR composite threshold of 1.17 and a centiloid threshold of 18 using the receiver operating characteristic (ROC = 0.99 for each; see Figure S2).

### 2.3 Plasma methods

At each main cognitive study visit, samples of blood were acquired whenever possible and processed as previously described^16^, ^17^ and stored at −80°C. Banked EDTA plasma samples were assayed to determine pTau217 concentration using the ALZpath single molecule array (Simoa) assay (ALZpath, Carlsbad, CA) on a Quanterix HD‐X instrument (Quanterix, Billerica, MA) running in the Wisconsin ADRC Biomarker Core. The assay itself was described previously by Ashton et al.^18^

### 2.4 Neuropsychological assessment and cognitive status

Participants in WRAP completed a comprehensive cognitive battery at each visit, including tests of memory, executive function, language ability, and other aspects of cognitive function, alongside self‐ and study partner‐based measures of everyday functioning.^19^ Based on these measures, participant cognitive status at each visit was determined via multidisciplinary consensus conference review.^20^ Similarly, participants in the ADRC completed a comprehensive cognitive battery as part of the Uniform Data Set (UDS, versions 2 and 3) in addition to select tests that were in common with WRAP. Participant cognitive status after each ADRC visit was also determined via consensus conference review. For both cohorts, the cognitive status determination was blind to biomarker information and validated diagnostic criteria were used to identify MCI^21^ and dementia.^22^ Given that cognitive assessments began in 2001 (WRAP) and 2009 (ADRC) and tau PET scans began in 2017, cognitive evaluations included in this study were predominantly completed before tau PET.

For this study, the primary cognitive outcome was a modified version of the PACC; PACC composites have been shown by others^23^ and our group^24^, ^25^ to be sensitive to AD‐related preclinical decline. The modified PACC included a measure of learning (the Rey Auditory‐Verbal Learning Test, sum of Trials 1‐5; AVLT);^26^ a separate measure of delayed recall (the Wechsler Memory Scale‐Revised^27^ Logical Memory II story A recall raw score or, after UDS3 was implemented in the ADRC, the cross‐walked equivalent score on the Craft Story delayed recall;^28^ and a measure of executive function common to both cohorts (the Trail Making Test part B time to completion; max 300 s).^29^ Tests were combined to create the PACC by rescaling each test and computing an unweighted average, scaled such that first observations in CU individuals were distributed ∼*N*(0,1) with higher scores representing better performance on each test and the PACC composite. For additional details on the two cohorts’ test batteries, harmonization, and cognitive composites, see reference.^25^

### 2.5 Statistics

All analyses were performed using R 4.4.0^30^ and the R‐packages lme4^31^ and emmeans^32^ to conduct the linear mixed models and post‐hoc comparisons, respectively. Sample characteristics including sex, age at amyloid and tau scans, amyloid status, amyloid burden, baseline plasma pTau217, baseline PACC performance, baseline cognitive status, and years of cognitive follow‐up were compared across the four tau categories (T−, T+_Neo_, T+_MTL_, and T+_MTL&Neo_) using analyses of variance (ANOVAs) or chi‐squared tests where appropriate for each variable; significant group differences were followed by pairwise comparisons to further describe how tau groups differed.

We used mixed effects models for both our cognitive and biological longitudinal outcomes of interest (i.e., PACC and plasma pTau217, respectively). In both retrospective analyses, we included the four group tau status as a categorical fixed effect (reference category = T−), and we incorporated random intercepts per participant.

For the cognitive analysis, we used the longitudinal PACC scores from each participant as the dependent variable. We included as fixed effects the tau status, linear and quadratic terms for the age at PACC assessment, as well as the interaction between tau status and the linear age term. Additionally, we also incorporated covariates for sex (female = 0, male = 1), education level (0 = no bachelor's degree [BA], 1 = BA or more), and practice. This practice covariate was a continuous predictor representing the number of times a participant was exposed to the cognitive test. It can take a value ranging from 0 (baseline) to 6 (representing 7–11 since a model using a number of exposures as a categorical variable showed plateauing of practice effects). Furthermore, since we observed a significant interaction between the tau status and the age predictor, we estimated marginal means for each tau group at ages 65, 70, 75, and 80 and performed paired comparisons between tau groups (using Tukey's adjustment), reporting the mean difference (*ΔM*) of the marginal effects at these ages.

For the plasma pTau217 analysis, we used the longitudinal concentration levels as the dependent variable. The analysis utilized ptau217 measures obtained from samples before tau PET in order to investigate early disease trajectories in soluble tau relative to presumed later‐stage fibrillar tau burden,^33^ operationalized by tau PET visual rating. In this model, instead of age at plasma visit, we operationalized time as relative to the earliest FQT scan (FQT distance = age at plasma visit–age at earliest FQT scan), to evaluate differences in the trajectories from each group relative to the time when the first tau PET exam was performed. We included as fixed effects the tau status, linear and quadratic terms for the FQT distance, as well as the interaction between tau status and the linear FQT distance. We additionally incorporated the age at tau scan, centered at 65, as a covariate. Finally, since we also observed a significant interaction between the tau status and the FQT distance, we estimated the marginal means for the pTau217 levels of each tau group at 8, 4, and 0 years from the first FQT PET, and then we performed paired comparisons between groups (also using Tukey's adjustment).

We also ran the cognitive and biofluid mixed effects models, subdividing the Tau groups by their amyloid status, resulting in eight groups instead of four: (A−/T−, A−/T+_MTL_, A−/T+_Neo,_ A−/T+_MTL&Neo_, A+/T−, A+/T+_MTL_, A+/T+_Neo,_ A+/T+_MTL&Neo_), using the same set of covariates we implemented before for each of them. Since we also observed a significant interaction between PET status and the time variables, we estimated marginal means for each group and reported the paired comparisons between groups, corrected for multiple comparisons too. In exploratory analyses, we compared incident transition rates across groups, where the incident transition was coded as moving from baseline CU to MCI or dementia at the last visit or baseline MCI to dementia at the last visit (Table S1).

## 3 RESULTS

Table 1 summarizes sample characteristics overall and by tau group for those included in the cognitive analyses. For the cognitive trajectories, there were 728 participants (573 (78.7%) T−; 25 (3.4%) T+_Neo_; 33 (4.5%) T+_MTL_; and 97 (13.3%) T+_MTL&Neo_). The average (standard deviation [SD]) age was 67.9 (7.4) years at the most recent tau scan, and 59.6 (7.5) years at the PACC3 baseline with 0.8 (2.0) years between the last cognitive assessment and the tau scan.

Overall, 221 (30.4%) of the participants were A+. The A+ proportions varied across groups: 95 (16.6%) in T−; 14 (56.00%) in T+_Neo_; 22 (66.7%) in T+_MTL_; and 90 (92.8%) in T+_MTL&Neo_. Additionally, the tau groups differed by age at cognitive baseline, baseline diagnostic frequency of impairment, and PACC score at the first visit. Specifically, we observed that participants in the T‐ group had lower age at cognitive baseline and higher PACC scores at baseline, relative to the T+_MTL&Neo_ group. The T+_MTL&Neo_ group showed a higher proportion of amyloid‐positive individuals compared to the other groups and a lower proportion who remained unimpaired at the most recent evaluation (48 of 97 were CU which is 49.48%, down from 80% CU at a prior baseline; see the diagnostic transition matrix table in the supplementary material). We did not see differences in sex composition, education level, or in number of years of cognitive follow‐up.

In exploratory analyses, the overall incident transition rate was 67(8.9%), including 57(7.6%) who transitioned from baseline CU to MCI or dementia and 10(1.3%) who transitioned from baseline MCI to dementia. The incident transition rates in each group were: T− 22(3.7%); T+ _MTL_ 3(9.2%); T+ _Neo_ 4(16%); and T+ _MTL&Neo_ 38(38.4%)_._ Fisher's exact tests showed these rates differed significantly across the four groups (*p* < 0.001). Follow‐up pairwise comparisons indicated that the T+_MTL&Neo_ group had significantly more participants transitioning into a worse cognitive status compared to T− (*p* < 0.001) and MTL (*p *= 0.006). We did not find significant differences between the other groups.

### 3.1 Cognitive trajectories

Model output for the retrospective PACC mixed effects model examining tau group by age interactions is provided in Table 2 (PACC model). Both the quadratic age term and the interaction between tau group and age were significant with parameter estimates indicating cognitive performance trajectories that become steeper (worse) at later ages, particularly in the T+_MTL&Neo_ group.

**TABLE 2:** Model output for mixed models.

	PACC Model			pTau217 Model		
Predictor	Estimate	CI	*p*‐value	Estimate	CI	*p*‐value
(Intercept)	−0.47	−0.62 – −0.33	**< 0.001**	0.34	0.32–0.36	**< 0.001**
T+_Neo_	−0.20	−0.55 – 0.16	0.273	0.16	0.06–0.25	**0.001**
T+_MTL_	0.04	−0.28 – 0.36	0.800	0.24	0.16 –0.32	**< 0.001**
T+_MTL&Neo_	−0.53	−0.73 – −0.34	**< 0.001**	0.64	0.59–0.71	**< 0.001**
Time^a^	−0.05	−0.06 – −0.04	**< 0.001**	0.01	0.01–0.01	**< 0.001**
Time^2^ ^a^	−0.01	−0.01 – −0.01	**< 0.001**	0.01	0.01–0.01	**0.002**
Sex (Male)	−0.47	−0.61 – −0.33	**< 0.001**			
Practice	0.13	0.11 – 0.15	**< 0.001**			
Education level (≥ BA)	0.53	0.39 – 0.67	**< 0.001**			
Age at FQT^b^	0.01			0.01	0.01 – 0.01	**< 0.001**
T+_Neo_ x Time	0.01	−0.02 – 0.03	0.652	0.02	0.01–0.02	**0.001**
T+_MTL_ x Time	−0.04	−0.06 – −0.02	**< 0.001**	0.02	0.01–0.02	**< 0.001**
T+_MTL&Neo_ x Time	−0.09	−0.11 – −0.08	**< 0.001**	0.05	0.04–0.05	**< 0.001**
Random effects						
*σ* ^2^	0.28			0.02		
τ_00_ _participant_	0.71			0.04		
ICC	0.72			0.73		
*N *	728			693		
Observations	3492			2304		
Marginal R^2^/conditional R^2^	0.320/0.808			0.402/0.836		

Abbreviation: ICC, intraclass correlation coefficient; PACC, Preclinical Alzheimer's Cognitive Composite.

^a^ In the PACC, model time and time^2^ correspond to age and age^2^ centered at 65. In the pTau217 model, these predictors correspond to the time difference in years between the first FQT scan and the pTau217 measurement.

^b^ Centered at 65.

**FIGURE 3:**
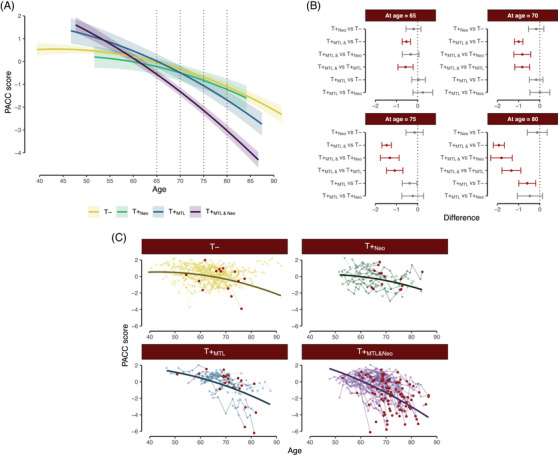
Estimated and observed cognitive trajectories among tau groups. (A) Predicted slopes across age for each of the tau groups. Solid lines represent the mean, and shaded regions show the 95% CI around it. Dotted lines highlight the time points (ages) selected for additional comparisons of simple slopes. (B) Paired comparisons between tau groups at ages 65, 70, 75, and 80. The dots and error bars represent the mean difference between groups with 95% CI, respectively. Significant differences are highlighted in red. (C) Spaghetti plots (dots and lines) represent the actual data observed in the different participants from each group. The solid lines on top correspond to the estimated slopes. For the T− group, we are only plotting 100 randomly selected individuals to improve visibility. The last observations from A+ participants are shown as red circles. For both panels A and B prediction lines are based on the following covariate values: sex = female, education = less than BA, practice = 0. BA, bachelor's degree; CI, confidence interval.

In Figure 3, we show both the observed and model‐estimated values for each group. In panel A we can see the estimated trajectories for each tau group (with sex = female, education = less than BA, practice = 0). Panel B shows the paired comparisons of each tau group's predicted PACC at ages 65, 70, 75, and 80. Finally, panel C displays the spaghetti plot of individual observed trajectories by tau group. As shown in Figure 3, panel B, none of the pairwise simple slope comparisons or predicted PACC levels between T− and T+_Neo_ differed significantly at any of the ages examined. In contrast, the average cognitive trajectory in T+_MTL&Neo_ participants deviated from the T− and T+_MTL_ groups at age 65 (T+_MTL&Neo_ vs. T−: Δ*M *= −0.53, *t*(724.79) = −5.32, *p* < 0.001; T+_MTL&Neo_ vs. T+_MTL_: Δ*M* = −0.58, *t*(737.27) = −3.16, *p* = 0.009), and all three T groups by age 70 (T+_MTL&Neo_ vs. T−: Δ*M* = −1.00, *t*(758.85) = −9.82, *p* < 0.001; T+_MTL&Neo_ vs. T+_Neo_: Δ*M* = −0.83, *t*(736.91) = −4.07, *p* < 0.001; T+_MTL&Neo_ vs. T+_MTL_: Δ*M* = −0.83, *t*(708.62) = −4.61, *p* < 0.001). At age 70, the average performance from T+_MTL&Neo_ (−1.32) was approximately 4.11 times worse than the performance from the T− group (−0.32). By age 80, these estimated differences were even larger (T+_MTL&Neo_ vs. T−: Δ*M* = −1.93, *t*(1578.92) = −14.55, *p* < 0.001; T+_MTL&Neo_ vs. T+_Neo_: Δ*M* = −1.81, *t*(1522.45) = −6.97, *p* < 0.001; T+_MTL&Neo_ vs. T+_MTL_: Δ*M* = −1.34, *t*(1367.01) = −5.90, *p* < 0.001). Additionally, at age 80, we also saw that participants in the T+_MTL_ group performed worse than T− participants (T+_MTL_ vs. T−: Δ*M* = −0.59, *t*(1373.07) = −2.89, *p *= 0.020).

### 3.2 Plasma pTau217 trajectories

For this analysis, a total of 693 participants were included (549 (79.2%) T−, 24 (3.5%) T+_Neo_, 32 (4.6%) T+_MTL_, and 88 (12.7%) T+_MTL&Neo_). The group characteristics were very similar to those observed in the PACC sample, (see Table S2 for details). We found differences in baseline pTau217 plasma levels, showing a higher baseline from the T+_MTL&Neo_ group compared to the others, and higher baseline levels from T+_MTL_ compared to T− participants. We did not observe differences in the number of years of plasma follow‐up or sex composition.

**FIGURE 4:**
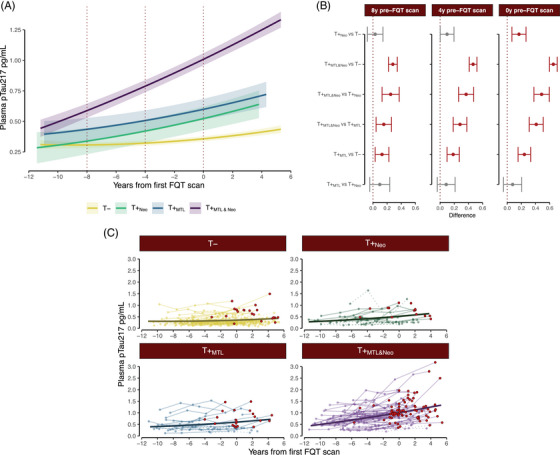
Estimated and observed plasma pTau217 trajectories among tau groups relative to years to/since tau scan. (A) Predicted slopes across time for each of the tau groups. Solid lines represent the mean, and shaded regions show the 95% CI around it. Dotted lines highlight the time points (years away from the FQT scan) selected for additional comparisons of simple slopes. (B) Paired comparisons between tau groups at 0, 4, and 8 years before the FQT scan. The dots and error bars represent the mean difference between groups with 95% CI, respectively. Significant differences are highlighted in red. (C) Spaghetti plots (dots and lines) represent the actual data observed in the different participants from each group. The solid lines on top correspond to the estimated slopes. For the T− group, we are only plotting 100 randomly selected individuals to improve visibility. A+ participants are shown as red circles. For both panels A and B, prediction lines are based on covariate values of FQT age = 65. CI, confidence interval; FQT, [F‐18]florquinitau.

Model output for the retrospective plasma pTau217 mixed effects model examining the tau group by time interactions is shown in Table 2 (pTau217 model). The significant quadratic time and significant tau group by time interaction (F(3,3469.39) = 68.26, *p *< 0.001) indicate that pTau217 plasma levels increased with time at different rates for the tau groups. Figure 4 depicts observed and estimated plasma pTau217. Panel A shows the estimated trajectories (and confidence intervals) by tau group (with centered FQT age = 0). In panel B, we display paired comparisons between tau groups, contrasting their estimated pTau217 levels at 8, 4, and 0 years pre‐FQT scan. Panel C shows the individual plasma pTau217 trajectories by tau group. Overall, we observed the estimated pTau217 levels at all time points were higher for T+_MTL&Neo_ participants compared to other tau groups. T+_MTL_ on the other hand showed significantly elevated pTau217 values compared to T− starting 4 years before the first FQT scan, and T+_Neo_ only showed higher levels than T− at the time of the FQT scan. We did not find differences between T+_Neo_ and T+_MTL_ at any of the timepoints reviewed.

Paired comparisons of estimated pTau217 levels indicated that participants in the T+_MTL&Neo_ group had higher plasma levels of pTau217 compared to the other groups at each of the times examined (including 0, 4, and 8 years before FQT scan). T+_MTL&Neo_ participants showed higher pTau217 levels compared to the other groups since 8 years prior to the FQT scan (T+_MTL&Neo_ vs. T−: Δ*M *= 0.28, *t*(1136.48) = 9.27, *p* < 0.001; T+_MTL&Neo_ vs. T+_Neo_: Δ*M* = 0.25, *t*(1058.72) = 4.30, *p* < 0.001; T+_MTL&Neo_ vs. T+_MTL_: Δ*M* = 0.18, *t*(1025.46) = 3.36, *p* = 0.004). These differences continued 4 years before the FQT scan (T+_MTL&Neo_ vs. T−: Δ*M* = 0.46, *t*(730.91) = 17.33, *p* < 0.001; T+_MTL&Neo_ vs. T+_Neo_: Δ*M* = 0.37, *t*(708.01) = 7.10, *p* < 0.001; T+_MTL&Neo_ vs. T+_MTL_: Δ*M* = 0.29, *t*(702.03) = 6.22, *p* < 0.001) and at the time of the FQT scan (T+_MTL&Neo_ vs. T−: Δ*M* = 0.64, *t*(752.26) = 23.96, *p* < 0.001; T+_MTL&Neo_ vs. T+_Neo_: Δ*M* = 0.49, *t*(770.65) = 9.17, *p* < 0.001; T+_MTL&Neo_ vs. T+_MTL_: Δ*M* = 0.40, *t*(778.38) = 8.46, *p *< 0.001).

We observed that, 8 years before the first FQT scan, on average, the estimated pTau217 level for T+_MTL&Neo_ (0.59) was 1.93 times higher than the estimated average for T− (0.30), and by the time of the FQT scan, they reached pTau217 levels 2.80 times higher than those present in T− participants (1.00 vs. 0.36). T+_MTL_ participants also showed higher predicted average pTau217 levels compared to T− 4 years before the FQT scan (T+_MTL_ vs. T−: Δ*M *= 0.11, *t*(974.70) = 2.36, *p* = 0.087), which continued to be present at the FQT scan (T+_MTL_ vs. T−: Δ*M* = 0.24, *t*(781.98) = 5.61, *p* < 0.001). On the other hand, T+_Neo_ participants only showed significantly higher pTau217 levels compared to T− at the time of the FQT scan (Δ*M* = 0.16, *t*(772.48) = 3.23, *p *= 0.007). We did not see differences between T+_MTL_ and T+_Neo_ at any of the timepoints analyzed.

### 3.3 Stratifying by amyloid and tau status

**FIGURE 5:**
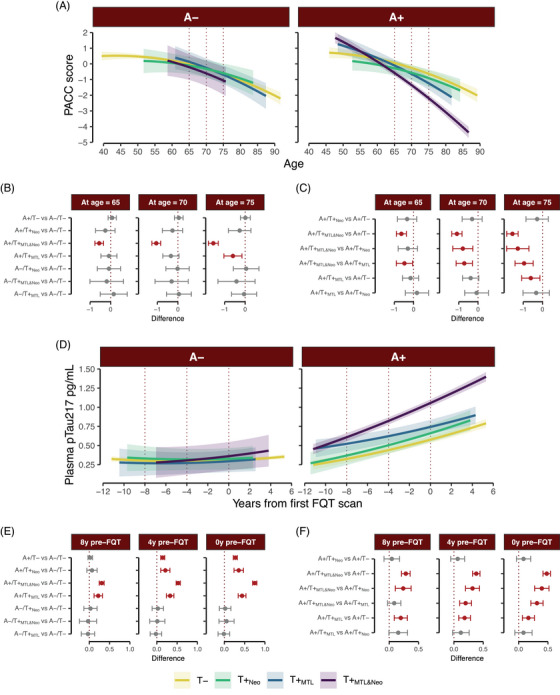
Cognitive and plasma pTau217 trajectories stratified by amyloid status. Significant A/T group*time interactions were followed with the depicted planned contrasts. (A) Estimated cognitive slopes across time for each of the tau groups. (B and C) Paired comparisons between tau groups. (D) Estimated plasma pTau217 slopes across time for each of the tau groups. (E and F) Paired comparisons between tau groups. Solid lines represent the mean, and shaded regions show the 95% CI around it. Dotted lines highlight the time points selected for additional comparisons of simple slopes. The dots and error bars represent the mean difference between groups with 95% CI, respectively. Significant differences are highlighted in red. CI, confidence interval.

Model output is provided in Table S3 and results are depicted in Figure 5. Briefly, in both analyses, the A/T group by time interaction was significant. In follow‐up comparisons, we only saw differences in group trajectories in the A+ participants, and the overall patterns observed shifted a bit. Overall, both the A+/T+_MTL_ and A+/T+_MTL&Neo_ groups showed lower average cognitive scores compared to A+/T− group at age 75 (A+/T+_MTL_ vs. A+/T−: Δ*M* = −0.60, *t*(950.67) = −2.51, *p* = 0.012; A+/T+_MTL&Neo_ vs. A+/T−: Δ*M* = −1.56, *t*(994.84) = ‐10.3, *p* < 0.001). However, no differences were found between the A+/T+_MTL_ and A+/T+_Neo_ at any of the timepoints tested (Figure 5 A–C).

In the pTau217 analysis, all A+/T+ groups showed elevated pTau217 levels compared to the A−/T− group 4 years before their first FQT scan (A+/T+_MTL_ vs. A−/T−: Δ*M* = 0.33, *t*(652.82) = 6.93, *p* < 0.001; A+/T+_Neo_ vs. A−/T−: Δ*M* = 0.21, *t*(656.9) = 3.66, *p* < 0.001; A+/T+_MTL&Neo_ vs. A−/T−: Δ*M* = 0.53, *t*(691.48) = 19.92, *p* < 0.001). Among these A+ groups, participants in the A+/T+_MTL&Neo_ group had the highest pTau217 levels 4 years before their first FQT scan (vs. A+/T−: Δ*M* = 0.38, *t*(693.94) = 11.78, *p* < 0.001; vs. A+/T+_MTL_: Δ*M* = 0.2, *t*(660.1) = 3.87, *p* < 0.001; vs. A+/T+_Neo_: Δ*M* = 0.32, *t*(662.26) = 5.21, *p* < 0.001). Additionally, the A+/T+_MTL_ displayed elevated biomarker levels compared to the A+/T− group, 4 years before their first FQT scan (Δ*M* = 0.18, *t*(659.02) = 3.58, *p* < 0.001). We did not find significant differences in pTau217 levels between the A+/T− and A+/T+_Neo_ groups at any time point tested.

## 4 DISCUSSION

This study examined the meaningfulness of qualitative T+ ratings from FQT (also known as MK‐6240) PET imaging using a previously published visual read framework.^10^ We compared tau positivity groupings for associations with amyloid PET burden, with phospho‐tau217 trajectories and with cognitive trajectories. Several insightful findings were observed. The T+_MTL&Neo_ group was clearly most affected by all outcomes. In this group, 93% were concurrently amyloid PET‐positive with rather high amyloid burden (median centiloid of 75.9) and rather pronounced cognitive decline from non‐symptomatic or mildly impaired baseline that differed in rate of declining trajectory from the other tau groups and in the number of transitions to cognitive impairment by the time of the most recent evaluation (20% had MCI at cognitive baseline, while 51% were diagnosed with MCI or dementia at the most recent cognitive exam). Moreover, plasma pTau217 concentration levels and trajectories were strongly related to the tau group, again with the T+_MTL&Neo_ group showing higher levels and faster rate of accumulation than the other groups. The T+_MTL&Neo_ group also exhibited elevations at least 8 years before the positive FQT PET exam, which were significantly higher than the other groups at this prior time point.

The T+_MTL&Neo_ category likely represents an intermediate to advanced biological stage of AD.^33^ According to the revised biomarker criteria, an advanced biomarker stage would be expected to be accompanied by amyloid positivity to be considered AD. This is consistent what we observed in that 93% of T+_MTL&Neo_ cases were also A+ and had higher estimated centiloid values which corresponded to approximately 11.6 years of amyloid positivity.^34^ Moreover, when stratified by amyloid status, the findings became even more pronounced for the A+ group. The lower rates of A+ concurrence for T+_MTL_ (67% A+) and T+_Neo_ (56% A+) suggest that these may be less mature groups in terms of their progressive proteinopathy, but may also reflect limitations in the rating process. More longitudinal studies are needed to characterize the course of spatial tau progression relative to amyloid status.^6^

An additional interesting observation in the pTau217 data supports the notion that T+ in the MTL *or* neocortex are early stages of this progressive disease. Specifically, even though the T+_MTL&Neo_ group exhibited the highest concentration and rate of change in pTau217, the faster accumulation and higher levels of pTau217 in both the T+_MTL_ and T+_Neo_ groups (relative to the T− group) before the tau scan, combined with the highly replicable well‐known strong association of pTau217 with A+ status,^18^ indicate that many in these smaller T+ groups are at higher likelihood of transitioning to more advanced biomarker stages of AD in the future. This is anecdotally illustrated in the longitudinal FQT PET images from four participants in the A+/T+_MTL&Neo_ who transitioned from an A+/T+Neo status to A+/T+_MTL&Neo_ (Figure S3). However, a further longitudinal study is needed to fully describe this, and is the topic of ongoing work.

The fibrillar tau patterns observed with FQT PET were associated with significant cognitive decline over the prior 10 years of observation. In particular, a positive tau signal that included both the MTL and at least one neocortical area was strongly associated with more rapid cognitive change which was significantly different from the T− group by age 65 and the T+_MTL_ and T+_Neo_ groups by age 70. However, we also observed significant but less rapid cognitive decline in the T+ _MTL_ only group relative to the T− group. The plots show that cognitive decliners in this group were also A+. Older participants in the T+ _MTL_ group exhibited greater cognitive change at this lower level of tau burden, perhaps reflecting additional non‐AD pathology contributing to cognitive decline. However, diagnostically the T+ _MTL_ group was largely stable with only 3 of 33 transitioning. Overall, the T+_MTL_ group was heterogeneous in that not all were declining, and some did not yet exhibit amyloid positivity. These factors suggest this group represents a less advanced stage. After stratifying by amyloid status, the cognitive and ptau217 effects were more pronounced when amyloid was present, and not present at all when amyloid was not present.

While prior literature, which is largely ROI‐based, finds that tau PET signal typically^9^ but not always^12^, ^13^ follows the progression of neurofibrillary tangle progression stages described by Braak and colleagues,^35^, ^36^, ^37^ we observe that almost as many are T+ in the neocortex only as are T+ in the MTL only. We suggest that these groupings are intermediate and eventually progress/spread to the combined pattern of T+_MTL&Neo_. The results reported here suggest that categorizing neocortical tau alone as evidence of advanced disease may be insufficient. The visual rating framework for flortaucipir^11^ in contrast considers neocortical tau as an advanced pattern where signal was present in typical Braak 5 areas, but it does not consider tau burden in the MTL in the positivity rating. In the present report, we find that neocortical T+_Neo_ was associated with flatter cognitive and pTau217 trajectories relative to T+ _MTL&Neo_ in this largely asymptomatic or mildly affected cohort, likely because neocortical tau on its own may reflect an earlier phase of the disease, but also perhaps because cognitive outcomes chosen in research studies such as this one are weighted toward episodic memory, a domain that is dependent more so on MTL structures rather than heteromodal neocortical structures.

Limitations to this work include that this is a sample of convenience of generally highly educated mostly non‐Hispanic White volunteers. Efforts are underway to further diversify the cohorts using methods and perspectives recently outlined^38^, ^39^ and will be the topic of future reports. There were logistical constraints on the study that resulted in missingness of cognitive outcomes due to the stoppage of in‐person visits due to COVID‐19 and slow resumption of those in‐person visits. We allowed a 3‐year span for inclusion to accommodate this. We reasoned this was permissible since AD is typically a slowly evolving disease that takes approximately 20+ years on average to reach the stage of mild dementia from the time of amyloid onset.^40^ A limitation in the visual rating system itself is that it does not distinguish the specific areas of the neocortex involved, and it does not explicitly demarcate moderate to advanced tau burden as a discrete category, as recommended in the revised AD biomarker framework.^33^ However, the results presented here suggest that, when tau signal is present in both the MTL and the neocortex, it may in fact already be at least a moderate stage of disease burden.

To conclude, in this paper, we present evidence that the category of tau PET positivity denoted by signal that is present in both the MTL and neocortex represent an advancing pattern of AD pathobiology that is associated with very high concordant rates of amyloid PET positivity, increased plasma pTau217 concentration, and unambiguous cognitive decline over a prior period of observation. This pattern of MTL and neocortical tau binding has research and clinical implications as these individuals are exhibiting clear disease progression of their proteinopathy and cognitive decline even though most did not exhibit dementia at their most recent FQT PET scan.

## CONFLICT OF INTEREST STATEMENT

H.Z. has served at scientific advisory boards and/or as a consultant for Abbvie, Acumen, Alector, Alzinova, ALZPath, Amylyx, Annexon, Apellis, Artery Therapeutics, AZTherapies, Cognito Therapeutics, CogRx, Denali, Eisai, LabCorp, Merry Life, Nervgen, Novo Nordisk, Optoceutics, Passage Bio, Pinteon Therapeutics, Prothena, Red Abbey Labs, reMYND, Roche, Samumed, Siemens Healthineers, Triplet Therapeutics, and Wave, has given lectures in symposia sponsored by Alzecure, Biogen, Cellectricon, Fujirebio, Lilly, Novo Nordisk, and Roche, and is a co‐founder of Brain Biomarker Solutions in Gothenburg AB (BBS), which is a part of the GU Ventures Incubator Program (outside submitted work). B.B.B. has consulted for New Amsterdam Pharma, Cognito Therapeutics, Merry Life Biomedical, and is co‐founder of Cognovance (outside submitted work). S.C.J. has served on scientific advisory boards for ALZPath and Enigma Biomedical. The following authors reported no financial or non‐financial disclosures: R.E.R.R., K.A.C., R.W., N.A.C., E.M.J., M.B., O.M., M.W., O.C.O., L.R.C., M.Z., S.A., B.T.C., T.J.B., L.E., and R.E.L. Author disclosures are available in the Supporting Information.

## CONSENT STATEMENT

The study procedures received approval from the University of Wisconsin‐Madison Institutional Review Board and were conducted in compliance with the World Medical Association Declaration of Helsinki. All subjects provided informed consent.

## Supporting information



Supporting Information

Supporting Information
