# Supplementary material for: Visual read of [F‐18]florquinitau PET that includes and extends beyond the mesial temporal lobe is associated with increased plasma pTau217 and cognitive decline in a cohort that is enriched with risk for Alzheimer's disease
Source: Alzheimers Dement. 2024 Nov 19;21(2):e14406. doi: 10.1002/alz.14406 (PMC11848396; doi:10.1002/alz.14406)
Supplement: Supplementary file 2 — Supporting Information [file ALZ-21-e14406-s001.docx]

**Supplementary Materials**


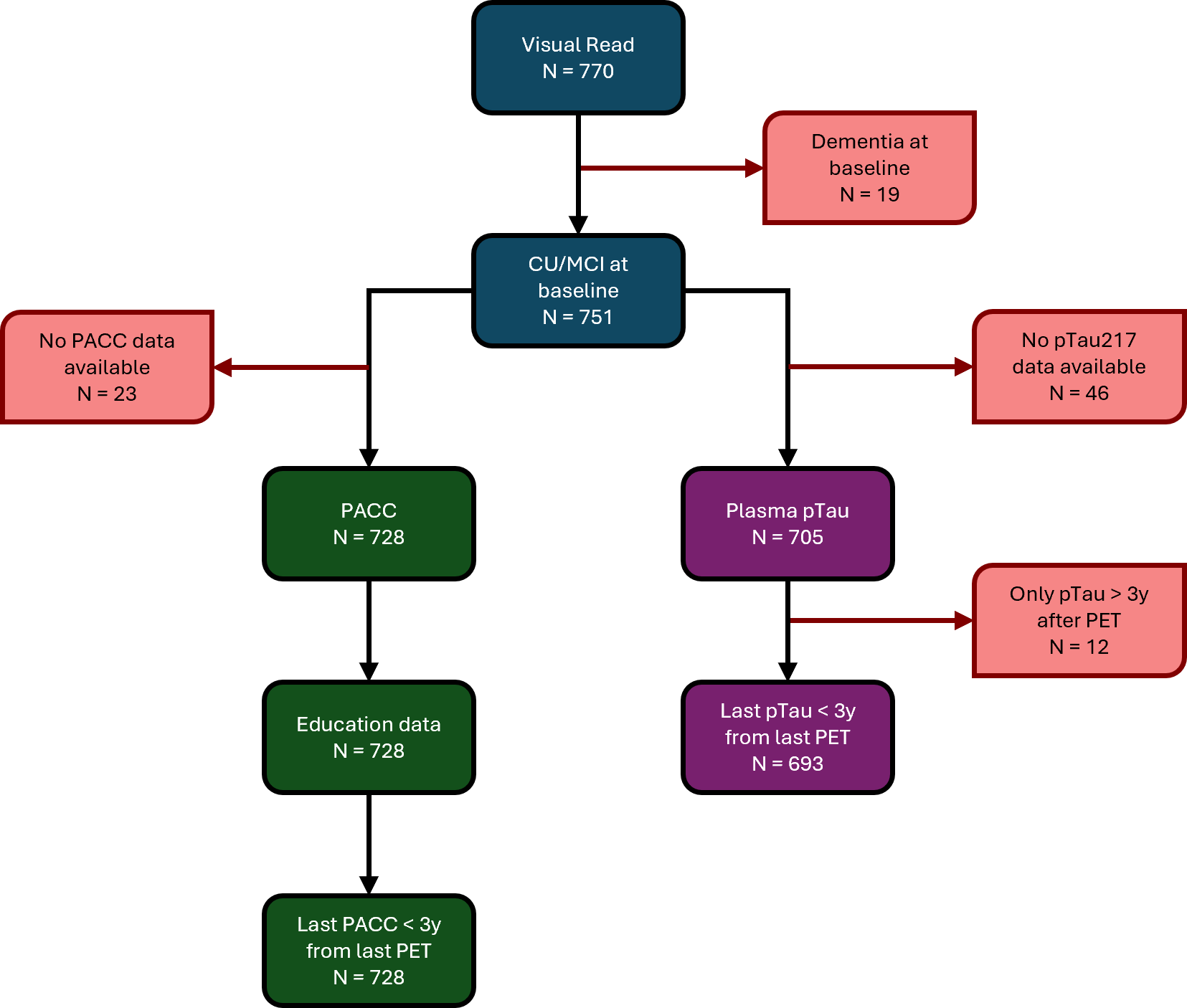


Figure S1. Consort diagram indicating eligibility filters and resulting n for the analysis reported. The base sample consisted of 770 participants with visual read from their PiB and FQT PET scans. From them, we kept 751 that were non-demented at their baseline cognitive evaluation. For the cognitive analysis (green) we kept 728 participants with PACC scores available, all of them fulfilled the rest of the requirements (education level available, PACC measurements less than three years after the last FQT scan). For the plasma pTau217 analysis, we kept 705 participants with pTau217 measurements available, and filtered out a final sample of 693 whose measurements occurred no more than three years after the last FQT scan.


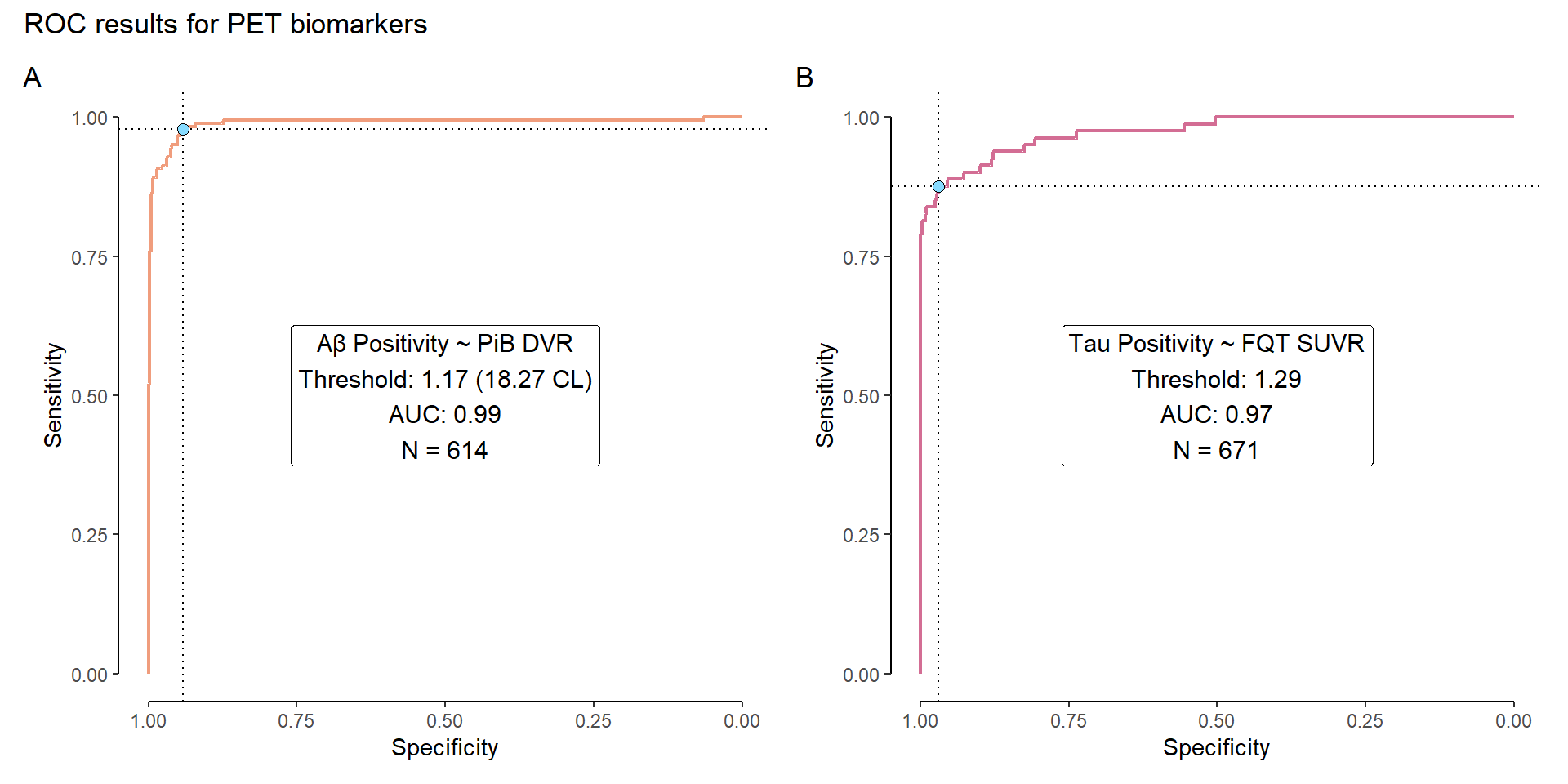


Figure S2. ROC fro2m amyloid positivity based on visual read against PiB DVR. The optimal threshold based on Youden’s method corresponds to an internal DVR composite of 1.17, which is equivalent to approximately 18 centiloids.


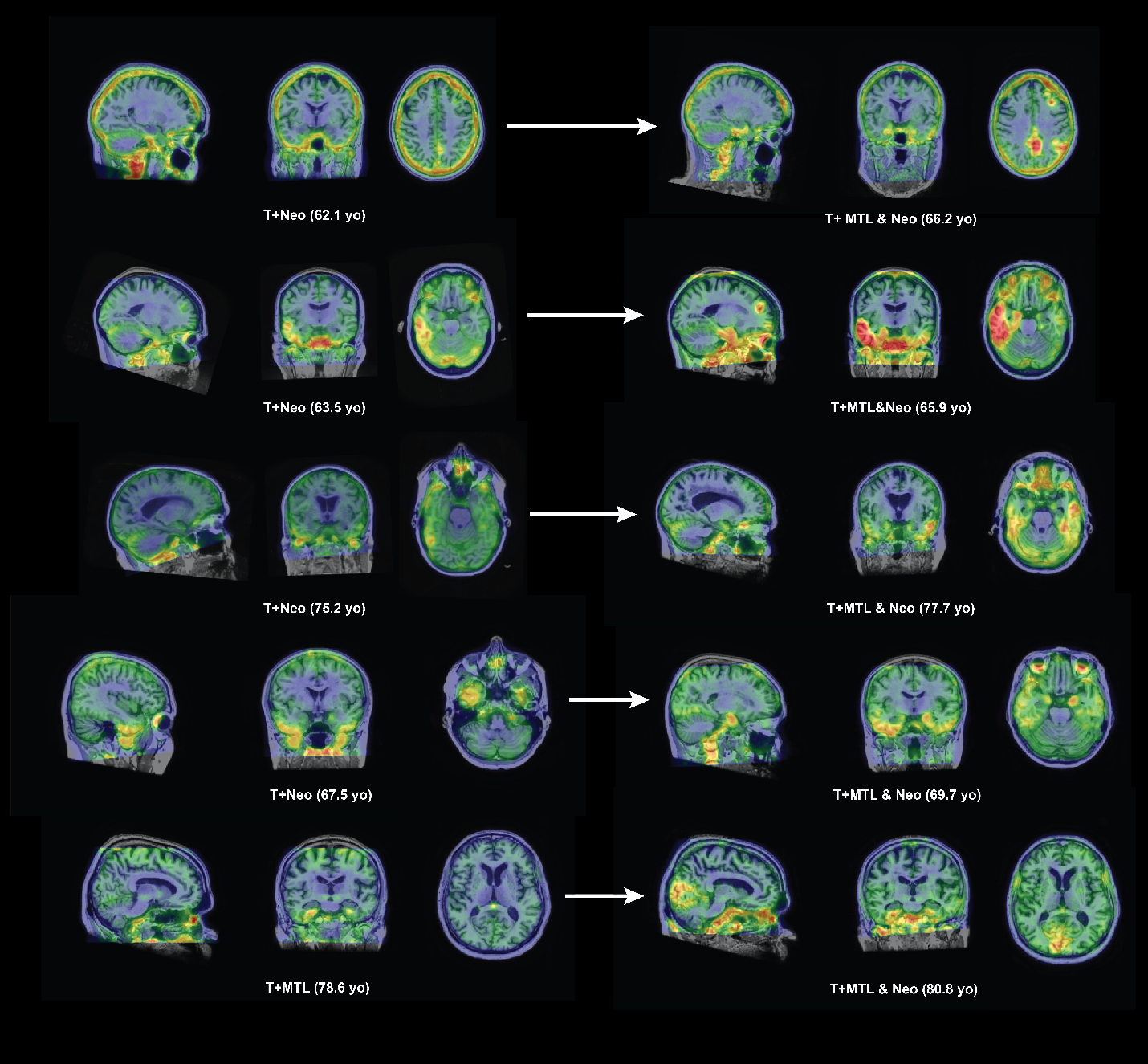


Figure S3. FQT PET scans showing transitions of tau status. Every row of images corresponds to a different participant. Images in the left side of the arrow represent the previous tau status, whereas those in the right side of the arrow show the PET scans after getting an updated tau status in the visual read. The first four participants showcase transitions from T+_Neo_ to T+_MTL&Neo_, the final participant exemplifies a case with a T+_MTL_ to T+_MTL&Neo_ instead.

Table S1. Transition matrix for cognitive diagnosis from prior baseline to the latest evaluation.

| ***Transition*** | ***Overall*** | ***T-*** | ***T+_Neo_*** | ***T+_MTL_*** | ***T+_MTL&Neo_*** |
| --- | --- | --- | --- | --- | --- |
| MCI - Dementia | 10 (1.3%) | 3 (0.51%) | 0 | 1 (3.0%) | 6 (6.1%) |
| MCI - MCI | 24 (3.2%) | 8 (1.35%) | 1 (4.0%) | 2 (6.1%) | 13 (13.1%) |
| MCI – CU | 3 (0.4%) | 3 (0.51%) | 0 | 0 | 0 |
| CU - Dementia | 9 (1.2%) | 0 | 0 | 0 | 9 (9.1%) |
| CU - MCI | 48 (6.4%) | 19 (3.2%) | 4 (16.0%) | 2 (6.1%) | 23 (23.2%) |
| CU - CU | 657 (87.5%) | 561 (94.4%) | 20 (80.0%) | 28 (84.9%) | 48 (48.5%) |
| Note: percentages shown are column wise, representing the prevalence of those transitions within groups. | | | | | |

Table S1B. Incident transition matrix

| ***Transition*** | ***Overall*** | ***T-*** | ***T+_Neo_*** | ***T+_MTL_*** | ***T+_MTL&Neo_*** |
| --- | --- | --- | --- | --- | --- |
| CU – MCI/Dementia | 57 (7.6%) | 19 (3.2%) | 4 (16.0%) | 2 (6.1%) | 32 (32.3%) |
| MCI - Dementia | 10 (1.3%) | 3 (0.5%) | 0 | 1 (3.0%) | 6 (6.1%) |
| No CI progression * | 684 (91.1%) | 572 (96.3%) | 21 (84.0%) | 30 (90.9%) | 61 (61.6%) |
| Notes: Percentages shown are column wise, representing the prevalence of those transitions within groups.  *This category represents all participants whose condition remained constant from their baseline evaluation. | | | | | |

Table S2: Sample characteristics of the pTau217 analysis, overall and by tau group:

| ***Variable*** | ***Overall*** | ***T-*** | ***T+_Neo_*** | ***T+_MTL_*** | ***T+_MTL&Neo_*** | ***p value*** |
| --- | --- | --- | --- | --- | --- | --- |
| pTau217 analysis, n (%) | 693 | 549 (79.2%) | 24 (3.5%) | 32 (4.6%) | 88 (12.7%) |  |
| Race, n (%) |  |  |  |  |  |  |
| American Indian or Alaskan Native | 18 (2.6%) |  |  |  |  |  |
| Asian | 1 (0.1%) |  |  |  |  |  |
| Black or African American | 54 (7.8%) |  |  |  |  |  |
| Other | 3 (0.4%) |  |  |  |  |  |
| White, hispanic | 6 (0.9%) |  |  |  |  |  |
| White, non-hispanic | 611 (88.2%) |  |  |  |  |  |
| Age at FQT scan | 67.8 (7.4) | 66.7 (7.3) | 71.3 (5.7)^+^ | 72.9 (7.3)^+^ | 71.2 (6.0)^+^ | **< .001** |
| Age at Aβ scan | 65.9 (7.9) | 64.9 (7.8) | 69.6 (7.3)^+^ | 71.8 (8.0)^+^ | 69.1 (6.6)^+^ | **< .001** |
| A+, n (%) | 210 (30.3%) | 93 (16.9%) | 14 (58.3%)^+^ | 21 (65.6%)^+^ | 82 (93.2%)^+^ | **< .001** |
| Baseline amyloid centiloids, median [Q1, Q3] | 5.94 [0.23, 17.74] | 3.44 [-0.65, 9.81] | 10.57 [2.00, 35.28] | 23.32 [8.18, 78.78]^+^ | 72.52 [36.08, 102.52]^++^ | **< .001** |
| Female, n (%) | 481 (69.4%) | 384 (70.0%) | 13 (54.8%) | 22 (68.8%) | 62 (70.5%) | = .431 |
| Age at baseline | 62.8 (7.9) | 61.9 (7.7) | 65.5 (8.0) | 67.2 (9.0)^+^ | 66.4 (7.1)^+^ | **< .001** |
| Years of plasma follow-up | 6.0 (3.9) | 6.0 (3.9) | 6.6 (3.5) | 6.4 (4.1) | 5.9 (3.9) | = .799 |
| Years between last plasma and FQT scan | 0.2 (1.6) | 0.2 (1.6) | 0.5 (1.8) | 0.5 (1.5) | 0.4 (1.7) | = .419 |
| Baseline pTau217 | 0.39 (0.26) | 0.32 (0.17) | 0.41 (0.26)^+^ | 0.48 (0.28)^+^ | 0.77 (0.35)^++^ | **< .001** |
| pTau217 A+ (> 0.46) n (%) at:  -9 < FQT distance < -7 (n = 211) | 45 (21.3%) | 17 (10.6%) | 2 (25.0%) | 6 (40.0%)^+^ | 20 (74.1%)^++^ | **< .001** |
| pTau217 A+ (> 0.46) n (%) at:  -1 < FQT distance < +1 (n = 477) | 138 (28.9%) | 69 (18.2%) | 5 (35.7%)^+^ | 9 (40.9%)^+^ | 55 (88.7%)^++^ | **< .001** |
| Adjusted baseline pTau217 (SEM)* |  | 0.32 (0.01) | 0.39 (0.04)^+^ | 0.46 (0.04)^+^ | 0.75 (0.02)^++^ | **< .001** |
| CU at baseline PACC, n (%) | 666 (96.1%) | 539 (98.2%) | 23 (95.8%) | 30 (93.8%)^+^ | 74 (84.1%)^+^ | **< .001** |
| CU at latest PACC, n (%) | 615 (88.7%) | 523 (95.3%) | 19 (79.2%) | 28 (87.5%)^+^ | 45 (51.1%)^++^ | **< .001** |
| *we show the marginal effects with covariates set as follows: age at FQT scan = 65.  ^+^ Different to T-; ^++^ Different to all groups | | | | | | |

Table S3: Model output for mixed models stratifying by amyloid status:

|  | **PACC Model** | | | **pTau217 Model** | | |
| --- | --- | --- | --- | --- | --- | --- |
| ***Predictor*** | ***Estimate*** | ***CI*** | ***p value*** | ***Estimate*** | ***CI*** | ***p value*** |
| (Intercept) | -0.47 | -0.62 – -0.33 | **<.001** | 0.29 | 0.27 – 0.31 | **<.001** |
| A-/T+_MTL_ | 0.15 | -0.51 – 0.81 | .656 | -0.01 | -0.16 – 0.13 | .876 |
| A-/T+_Neo_ | -0.08 | -0.65 – 0.48 | .773 | 0.02 | -0.12 – 0.16 | .780 |
| A-/T+_MTL&Neo_ | -0.19 | -1.01 – 0.63 | .649 | 0.05 | -0.13 – 0.24 | .564 |
| A+/T- | 0.07 | -0.12 – 0.27 | .462 | 0.27 | 0.22 – 0.32 | **<.001** |
| A+/T+_MTL_ | -0.08 | -0.47 – 0.31 | .674 | 0.44 | 0.34 – 0.53 | **<.001** |
| A+/T+_Neo_ | -0.26 | -0.73 – 0.21 | .283 | 0.35 | 0.24 – 0.47 | **<.001** |
| A+/T+_MTL&Neo_ | -0.56 | -0.77 – -0.35 | **<.001** | 0.75 | 0.70 – 0.80 | **<.001** |
| Time* | -0.05 | -0.06 – -0.04 | **<.001** | 0.01 | 0.01 – 0.01 | **<.001** |
| Time^2^* | -0.01 | -0.01 – -0.01 | **<.001** | 0.01 | 0.01 – 0.01 | **.001** |
| Sex (Male) | -0.47 | -0.61 – -0.33 | **<.001** |  | | |
| Practice | 0.13 | 0.11 – 0.15 | **<.001** |  | | |
| Education level (>= BA) | 0.53 | 0.39 – 0.67 | **<.001** |  | | |
| Age at FQT** |  | | | 0.01 | 0.01 – 0.01 | **.004** |
| Time x A-/T+_MTL_ | -0.02 | -0.06 – 0.02 | .297 | 0.00 | -0.01 – 0.02 | .717 |
| Time x A-/T+_Neo_ | 0.01 | -0.02 – 0.05 | .455 | 0.00 | -0.02 – 0.01 | .897 |
| Time x A-/T+_MTL&Neo_ | -0.02 | -0.08 – 0.04 | .451 | 0.01 | -0.01 – 0.03 | .217 |
| Time x A+/T- | -0.01 | -0.02 – 0.01 | .333 | 0.03 | 0.03 – 0.04 | **<.001** |
| Time x A+/T+_MTL_ | -0.05 | -0.08 – -0.02 | **<.001** | 0.03 | 0.02 – 0.04 | **<.001** |
| Time x A+/T+_Neo_ | -0.00 | -0.03 – 0.03 | .955 | 0.04 | 0.02 – 0.05 | **<.001** |
| Time x A+/T+_MTL&Neo_ | -0.10 | -0.11 – -0.09 | **<.001** | 0.06 | 0.05 – 0.06 | **<.001** |
| **Random effects** | | | | | | |
| σ^2^ | 0.28 | | | 0.02 | | |
| τ_00_ _participant_ | 0.71 | | | 0.04 | | |
| ICC | 0.72 | | | 0.71 | | |
| N | 728 | | | 652 | | |
| Observations | 3492 | | | 2162 | | |
| Marginal R^2^ / Conditional R^2^ | 0.320 / 0.808 | | | 0.502 / 0.857 | | |
| **In the PACC model time and time^2^ correspond to age and age^2^ centered at 65. In the pTau217 model, these predictors correspond to the time difference in years between the first FQT scan and the pTau217 measurement.*  ***Centered at 65* | | | | | | |
